# Draft genome sequences of *Bordetella parapertussis* clinical isolates during an increase in confirmed cases from Colombia, 2023

**DOI:** 10.1128/mra.00672-24

**Published:** 2025-02-05

**Authors:** Efraín Montilla-Escudero, Fabiola Rojas-Baquero, Valentina Bonilla-Bravo, Nathalia Vargas-Florez

**Affiliations:** 1 Grupo de Microbiología, Subdirección Laboratorio Nacional de Referencia, Dirección de Redes en Salud Pública, Instituto Nacional de Salud, Bogotá, Colombia; SUNY College of Environmental Science and Forestry, Syracuse, New York, USA

**Keywords:** *Bordetella parapertussis*, whooping cough like disease, NGS

## Abstract

We report three *Bordetella parapertussis* genome sequences obtained from national pertussis surveillance in Colombia during an increase in confirmed cases not linked to outbreaks

## ANNOUNCEMENT

*Bordetella parapertussis* causes a whooping cough-like illness with milder symptoms than pertussis (1). Since 1997, the Microbiology Group has monitored *B. pertussis* and *B. parapertussis* via culture, incorporating PCR in 2011 (2). Laboratory data revealed a 2,750% surge (*P* < 0.05) in *B. parapertussis* detection in 2023 (*n* = 57) compared with 2022 (*n* = 2), 2021, and 2020 (*n* = 0). The cause remains unclear, though a similar rise occurred in Chile in 2023 (3).

The cases, from Bogotá institutions, involved a 15-year-old girl and two 1-year-old infants, all fully vaccinated. Diphtheria-Tetanus-Pertussis (DTP) vaccines lack proven efficacy against *B. parapertussis (4*). Two patients had bronchitis; one infant showed apnea, stridor, vomiting, paroxysmal cough, and a 20-day cough treated with clarithromycin.

The strains were isolated from a nasopharyngeal swab sample using Regan–Lowe agar with cephalexin (40 µg/mL) and 10% horse blood. They were then sent to the Microbiology Group for species confirmation using Matrix-Assisted Laser Desorption/Ionization Time of Flight Mass Spectrometry (MALDI-TOF MS) and conventional tests (5). DNA was extracted from isolates grown aerobically at 37°C on Regan–Lowe agar (BD) for 48 h using the QIAamp DNA Mini Kit (Cat 51304) (QIAGEN, Hilden, Germany). Subsequently, multiplex real-time PCR was conducted to detect *pIS*1001, confirming the presence of *B. parapertussis* (6).

DNA libraries with a 200 bp paired-end insert size were prepared using the Nextera DNA Flex Library Preparation Kit (Illumina, USA) and sequenced on the MiSeq (Illumina) with at least 40-fold coverage. Raw reads were trimmed with Trimmomatic v0.39 (github.com/timflutre/trimmomatic) and assembled using SPAdes v3.14.0 (github.com/ablab/spades) under default parameters. Sequences were annotated automatically using NCBI PGAP v5 (7), with quality parameters detailed in Table 1. Antibiotic resistance genes were identified using CARD (https://card.mcmaster.ca/analyze) (8), and virulence genes were annotated with the VFDB database (http://www.mgc.ac.cn/VFs/main.htm) (Table 1) (9).

**TABLE 1:** Characteristics of *Bordetella parapertussis,* 2023^*a*^^,^^*m*^

Parameter	Isolates		
	320001750	320001751	320001806
NCBI Biosample	SAMN38746480	SAMN38746481	SAMN38746482
Number of raw reads (bp)	905,057	1,204,205	1,179,600
Coverage (X)	47.98	62.789	62.394
N50 (bp)	52,690	57,113	41,541
Genome size (bp)	4,707,904	4,710,683	4,706,379
%GC content	68.13	68.14	68.12
Number of contigs	203	197	262
Total no. of genes	4,596	4,588	4,625
Total no. of coding sequences	4,539	4,531	4,568
No. of complete rRNAs (5S, 16S, 23S)	3	3	3
No. of tRNAs	50	50	50
No. of noncoding RNAs	4	4	4
Total no. of pseudogenes	231	228	226
No. of insertion sequences IS1001 (identity >99.0%)	54	54	50
No. of insertion sequences IS1002 (identity >98.6%)	10	10	10
No. of plasmids	0	0	0
No. of phage score >90 (Phaster)	0	0	0
Sequence type	19	19	19
Virulence genes	**Pertussis toxin^*b*^** (p*tx*A*, ptx*B*, ptx*C*, ptx*D*, ptx*E) (*ptl*A*, ptI*B*, ptI*C*, ptI*D*, ptI*E*, ptI*F*, ptI*G*, ptI*H*, ptl*I)^*c*^ **Fimbriae^*d*^** (*fim*B*, fim*C*, fim*D)^*e*^ **Filamentous hemagglutinin^*f*^** (*fha*B*, fha*C)^*g*^ **RTX toxin^*h*^** (*cya*A*, cya*B*, cya*C*, cya*D*, cya*E)^*i*^ **Pertactin^*j*^** (*prn*)^*k*^	**Pertussis toxin** (p*tx*A*, ptx*B*, ptx*C*, ptx*D*, ptx*E) (*ptl*A*, ptI*B*, ptI*C*, ptI*D*, ptI*E*, ptI*F*, ptI*G*, ptI*H*, ptl*I) **Fimbriae** (*fim*B*, fim*C*, fim*D) **Filamentous hemagglutinin** (*fha*B*,fha*C) **RTX toxin** (*cya*A*, cya*B*, cya*C*, cya*D*, cya*E)	**Pertussis toxin** (p*tx*A*, ptx*B*, ptx*C*, ptxD, ptxE*) (*ptl*A*, ptI*B*, ptI*C*, ptI*D*, ptI*E*, ptI*F*, ptI*G*, ptI*H*, ptl*I) **Fimbriae** (*fim*B*, fim*C*, fim*D) **Filamentous hemagglutinin** (*fha*B*,fha*C) **RTX toxin** (*cya*A*,cya*B*,cya*C*,cya*D*, cya*E) **Pertactin** (*prn*)
Antibiotic resistance	**Tigecycline resistance** *rps*J **Efflux pump** **tetracycline resistance** MexAB-OprM^*l*^	**Tigecycline resistance** *rps*J **Efflux pump** **tetracycline resistance** MexAB-OprM	**Tigecycline resistance** *rps*J **Efflux pump** **tetracycline resistance** MexAB-OprM
cgMLST reference genome	**NC_002928.3** (**1,370 targets**)	**NC_002928.3** (**1,370 targets**)	**NC_002928.3** (**1,370 targets**)
cg MLST matches	**1328**	**1309**	**1316**
cgMLST allelic differences	**320001751 = 35** **320001806 = 10**	**320001750 = 35** **320001806 = 25**	**320001750 = 10** **320001751 = 25**
SNPs (Fastree)	**320001751 = 249** **320001806 = 22**	**320001750 = 249** **320001806 = 247**	**320001750 = 22** **320001751 = 247**

^*a*^ Colombia.

^*b*^ Genes present in both *Bordetella pertussis* and *B. parapertussis* (not expressed due to mutations in the promoter).

^*c*^ Genes of pertussis toxin (*ptx*) and toxin secretion (*ptl*).

^*d*^ Hair-like appendages aid bacterial adhesion, crucial for colonization and infection.

^*e*^ They encode fimbriae and the proteins for transport and assembly.

^*f*^ Filamentous protein that facilitates bacterial adhesion to epithelial cells in the respiratory tract and to immune cells

^*g*^ fhaB produces FHA; fhaC transports FHA to the bacterial surface.

^*h*^ Repeats in toxin damages host cells, alters immune function, and promotes tissue invasion.

^*i*^ Encodes the adenylate cyclase-hemolysin protein and these genes are responsible for the modification, activation, and secretion

^*j*^ Highly immunogenic, cell adhesion via RGD interaction.

^*k*^ Pertactine gen.

^*l*^ RND-type efflux pump (Resistance-Nodulation-Division).

^*m*^ The bold text refers to the main names of the genes in the sections on virulence genes and antimicrobial resistance.

The insertion genes IS*1001* and IS*1002* were also identified in the genomes with Nucleotide Blast v2.15.0; only results with identity >98.95% were accepted (10).

All isolates were identified as ST19 by MLST (11); however, it was necessary to conduct a core genome annotation using the tool bigsd.pasteur.fr to verify the distance between the isolates, as MLST was not discriminatory.

All isolates shared >95% matched cgMLST targets, with 10–35 allelic differences, indicating a likely circulating clone despite no epidemiological or spatial links between isolates 320001750 and 320001806. SNP analysis (22 SNPs) confirmed high relatedness using CSIPhylogeny (default parameters, Table 1) (12). These isolates averaged 50 IS*1001* copies, significantly higher than the 20 reported in other studies (13), which may affect genome rearrangement or gene regulation.

## Data Availability

Raw sequence reads of *B. parapertussis* was deposited in Sequence Read Archive (SRA) SRR27160578, SRR27160579 and SRR27160580, BioProject accession number PRJNA1049066. Draft genomes sequences of the three B. parapertussis isolates have been deposited in GenBank database under accession numbers JAXUBE000000000, JAXUBF000000000, and JAXUBG000000000.
